# Multiomics Study of Gut Bacteria and Host Metabolism in Irritable Bowel Syndrome and Depression Patients

**DOI:** 10.3389/fcimb.2020.580980

**Published:** 2020-10-29

**Authors:** Congmin Xu, Qiong Jia, Lu Zhang, Zhe Wang, Shiwei Zhu, Xiaoqi Wang, Yixuan Liu, Mo Li, Jingjing Zhang, Xiangqun Wang, Jindong Zhang, Qinghua Sun, Kun Wang, Huaiqiu Zhu, Liping Duan

**Affiliations:** ^1^ Department of Biomedical Engineering, College of Engineering, and Center for Quantitative Biology, Peking University, Beijing, China; ^2^ Department of Gastroenterology, Peking University Third Hospital, Beijing, China; ^3^ Department of Psychiatry, Institute of Mental Health, Peking University, Beijing, China

**Keywords:** trehalose and maltose hydrolase, fucose permease, Ure, *E. coli*, *E. cloacae*

## Abstract

**Background and Aims:**

Irritable bowel syndrome (IBS) and depression have high tendencies of comorbidity. In particular, diarrhea-predominant IBS (IBS-D) and depression exhibit similar fecal microbiota signatures, yet little is known about their pathogenic mechanism. Here, we propose that the differences in structure and composition of IBS-D and depression gut microbiota give rise to different downstream functions, which lead to distinct clinical phenotypes *via* host metabolism and further influence the interaction of brain–gut axis.

**Methods:**

We performed multiomics study, including fecal metagenome-wide sequencing and serum metabolomics profiling in 65 individuals with IBS-D (n=22), depression (n=15), comorbid patients (n=13), and healthy controls (n=15). We analyzed functional genes contributed by the primary genus and evaluated their correlations with clinical indices and host metabolites.

**Results:**

Metagenomic analysis revealed 26 clusters of orthologous groups of protein (COG) categories consisting of a total of 4,631 functional genes. Trehalose and maltose hydrolase (COG1554) and fucose permease (COG0738) were the most relevant and enriched functional genes in the IBS-D patients; urease accessory proteins UreE (COG2371) was that in the depression patients. Context based genome annotation suggest that an alteration of *Escherichia coli* and *Enterobacter cloacae* in IBS-D and depression respectively may be responsible for the enrichment described above. Correlation with host metabolites, such as maltotriose and isomaltose in carbohydrate metabolism and anandamide in neuroactive metabolism, drew further connections between these findings.

**Conclusions:**

These changes led us to propose a connection between genomic signatures and clinical differences observed in IBS-D and depression. Our findings provide further insights into the involvement of gut microbiota in diseases related to brain–gut disorder.

## Introduction

Irritable bowel syndrome (IBS) is defined as a disorder of brain–gut interactions according to the updated Rome IV criteria (Ford et al., 2017; Lacy et al., 2018). IBS and depression exhibit high tendencies of comorbidity (Labus et al., 2017; Chang et al., 2018). Accumulating evidences have suggested microbiota as one of the most important contributing factors to not only IBS (Moloney et al., 2016; Powell et al., 2017) but also depression (Valles-Colomer et al., 2019). Interestingly, our previous studies on human fecal samples identified striking similarities between the compositions of gut microbiota in diarrhea-predominant IBS (IBS-D) and depression patients (Liu et al., 2016).

In our previous study, using 454 pyrosequencing of 16S rRNA, we categorized gut microbiota from all patients into three distinct types—*Bacteroides* dominant (type I), *Prevotella* dominant (type II), and non-dominant, healthy-like microbiota (type III). Most IBS-D (85%) and depression (80%) patients exhibited type I and type II profiles with comparable compositions, whereas majority of the healthy controls (95%) exhibited type III profile and a greater bacterial diversity according to the Shannon index. Furthermore, type I and II profiles were associated with high colonic mucosa inflammation levels and IBS symptom severity. Nevertheless, the question as to whether similar bacterial compositions manifested different clinical presentations remains unclear. To distinguish the pathogenic mechanisms of these two diseases, detailed assessment of the microbial functions is required.

Microbial functions may differ greatly even in the same species, resulting in distinct metabolic products and modes of interaction both within the gut microbiota and between the host-gut ecosystems, causing various pathophysiological shifts (Zhang and Zhao, 2016). Metagenome-wide association study (MWAS) enables higher resolution investigation into the functions of gut microbiome than 16S rRNA amplicon sequencing (Lloyd-Price et al., 2017). In order to find out why IBS-D and depression owned similar bacterial compositions but different clinical presentations, the aim of the present study is trying to find out the differences between IBS-D and depression from functional genomics of gut microbiota, as well as the fecal short-chain fatty acids (SCFA) and host serum metabolomics. Furthermore, the relationship between those multiomics parameters and the clinical manifestation were analyzed. We performed multiomics study, including MWAS along with fecal SCFAs quantification and serum metabolomics profiling in both diseases and the comorbid (COMO) cohort. Our results highlighted different functional genes in these three groups and their potential interactions with the host.

## Materials and Methods

### Study Recruitment and Study Design

Adult subjects aged 18–65 years with IBS-D and/or depression were recruited prospectively. Patients fulfilling Rome III criteria for IBS-D were recruited at the Outpatient Department of Gastroenterology of Peking University Third Hospital, while patients meeting the Mini-International Neuropsychiatric Interview (MINI) DSM-IV criteria for depression were recruited at the Peking University Outpatients Department of the Institute of Mental Health. Healthy volunteers were recruited separately as controls. All subjects were evaluated by both a gastroenterologist and a psychologist to ensure accurate grouping. Patients who met criteria for both diagnoses were recruited as the comorbid (COMO) cohort.

Exclusion criteria included organic gastrointestinal or systemic diseases such as inflammatory bowel diseases or diabetes mellitus; concurrent infections of the respiratory, digestive, or urinary systems; history of abdominal surgery other than appendicitis; use of antibiotics or antidepressant during the previous month; use of probiotics, laxatives, or anti-diarrheal drugs for more than 3 days during the previous 2 weeks; and pregnancy or lactation. Colonoscopy was performed on every subject to ensure the absence of organic gastrointestinal diseases and sigmoid mucosal biopsies were taken.

All subjects gave their informed consent for inclusion before they participated in the study. The study was conducted in accordance with the Declaration of Helsinki, and the protocol was approved by the Ethics Committee of Peking University Health Science Center (No. 2013-112).

### Assessment of Symptom Severity

IBS symptom severity was assessed using the validated IBS-Symptom Severity Scale (IBS-SSS) (Francis et al., 1997) and depression severity was evaluated using the Zung’s self-rating depression scale (SDS) (Yin et al., 2015).

### Sample Collection and Immunohistochemistry

Fecal samples of the MWAS in this study were the same set of samples as the 16S rRNA sequencing which we used in reference 8. Fecal samples were collected before colonoscopy and stored at −80°C until use. Details of fecal samples collection described in the **Supplementary Materials and Methods**. Blood samples were collected before colonoscopy in heparinized anticoagulative tubes and then centrifuged 1,000 *g* for 5 min to separate plasma. Sigmoid biopsied mucosal tissues were obtained during colonoscopy and immunohistochemistry staining was performed as described in **Supplementary Materials and Methods**.

### Detection of Systematic Inflammation Using ELISA

To measure systematic inflammation levels, plasma cytokines including MCP-1, IL-6, IL-10 and IL-12 were measured using a Human Magnetic Luminex Screening Assay (11 PLEX) (R&D LXSAHM-11). More details are given in the **Supplementary Materials and Methods**.

### Quantification of Fecal SCFAs

Seven SCFAs (formate, acetate, propionate, butyrate, isobutyrate, valerate, and isovalerate) were measured using an isotope-labeled chemical derivatization method on liquid chromatography-tandem mass spectrometry with slightly modified parameters (Kelley et al., 2010). More details described in the **Supplementary Materials and Methods**.

### Metagenomic Sequencing

Details of methods for DNA extraction, library construction and sequencing were described in the **Supplementary Materials and Methods**. Qualified DNA samples were sheared into smaller fragments by nebulization. The overhangs resulted from fragmentation are converted into blunt ends using T4 DNA polymerase, Klenow fragment, and T4 polynucleotide kinase. An adenine (A) was added to the 3’ end of the blunt phosphorylated DNA fragments before ligated to adapters. Short fragments were removed using Ampure beads. All samples were sequenced on the Illumina HiSeq™ platform.

### Metagenomic Data Processing and Statistical Analysis

To carry out the metagenomic analysis, sequenced raw data with length of 90 or 150 bp per read were first pretreated by Quake (Schmieder and Edwards, 2011) and Prinseq (Lai et al., 2015) to filter out reads with low quality. The validated reads were assembled into contigs utilizing InteMAP (Liu et al., 2013), an integrated metagenomic assembly pipeline specially designed for next-generation sequencing (NGS) short reads. MetaGUN (Hu et al., 2009) and MetaTISA (Galperin et al., 2015) were then used successively for metagenomic gene prediction and gene TIS refinement. The parameters were set as default. We used BLAST (Friedman and Alm, 2012) to functionally annotate genes by searching against Clutsters of Orthologous Groups of proteins (COG) database (Altschul et al., 1990; Zhang and Zhao, 2016), where the e-value threshold was set to 10^−3^. Based on the functional annotation result principal component analysis (PCA) and partitioning around medoids (PAM) were applied to further clarify individual differences. Permutational one-way ANOVA was used to identify the functions that were significantly different among groups. Spearman’s correlation was used to test correlations between microbial gene function and host metabolites or symptom severity.

### Non-Target Metabolomics

All serum samples were acquired by the LC-MS system followed machine orders. Non-target metabolomics of serum was performed as described in the **Supplementary Materials and Methods**.

### Synthesis of Functional Co-Occurrence Networks

The human gut microbial community is a complex ecosystem. We elucidated symbiotic interactions among functions in the gut microbiome. We used SparCC (Friedman and Alm, 2012) to calculate Spearman correlation coefficients and permutational p-values (adjusted by false discovery rate, FDR) between each pair of functions. Once the two values reached the given thresholds, an edge was generated in the functional co-occurrence network, wherein the nodes represent functions and edges represent association between two functions. The result visualized using Cytoscape (Shannon et al., 2003). More details were described in the **Supplementary Materials and Methods**.

### Identification of Biomarkers

We further infer biomarkers as those functions with significantly different abundance in different groups (permutational one-way ANOVA, *P* < 0.01 with 1000 randomizations) and also correlated with clinical indices (Spearman correlation with *P* < 0.05). IBS-related clinical indices include symptoms (IBS-SSS, abdominal pain, onset frequency of pain, bloating, satisfaction to bowel habit, interference to quality of life, maximum bowel movement), visceral sensation (first sensation of need to defecate, urgency, pain threshold, maximum tolerance), fecal SCFA levels, and inflammation factors. Depression-related clinical index included SDS, and inflammation-related factors included SCFAs. We calculated Spearman correlations between each microbial function and clinical indices of samples in the corresponding group. Functions significantly correlated with at least one clinical index were selected as functional biomarkers. Biomarkers for different groups were those functions reached these above-mentioned criteria and with highest abundance in the corresponding group. With these biomarkers, we trained classification model using a machine learning algorithm (random forest) to discriminate the specific disease group from samples in the other groups.

## Results

### Demographic and Clinical Characteristics of IBS-D, Depression, COMO Patients, and Healthy Controls

**Figure 1:**
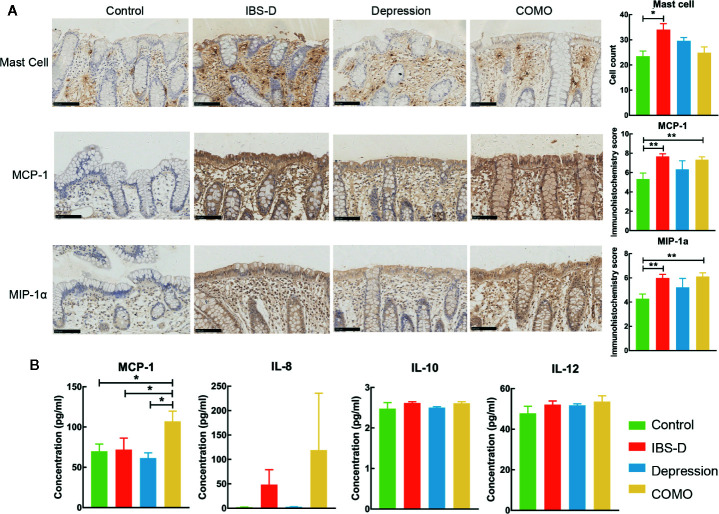
Cytokines of mucosal and plasma. **(A)** Immumohistochemical staining results of sigmoid mucosal mast cells, MCP-1 and MIP-1α. **(B)** Statistic of plasma MCP-1, IL-8, IL-10 and IL-12. In this figure, * represents P value <0.05 and ** represents P value <0.01.

The study cohort included 65 study subjects, with 22 IBS-D patients, 15 depression patients, 13 COMO patients and 15 healthy controls. Gender, age and BMI scores did not significantly differ across study groups (**Table 1**). IBS-D and depression symptom severity were assessed for all subjects. IBS-D patients and COMO patients had much severer abdominal symptoms than did depression patients and healthy controls, including abdominal pain, onset frequency of abdominal pain, abdominal bloating, and satisfaction with bowel habit and interference with quality of life. Depression and COMO patients had higher Zung’s self-rating depression scale (SDS) scores than IBS-D patients and healthy controls (**Table 1**). Because low-grade inflammation was observed in both IBS and depression, we measured sigmoid mucosal cytokines by immunohistochemistry and plasma cytokines using ELISA (**Figure 1** and **Supplementary Table 1**). In the colon mucosal, mast cell counts were significantly higher in IBS-D than in COMO patients and healthy controls, and MCP-1 and MIP-1α levels were significantly higher in IBS-D and COMO patients than in healthy controls. Only plasma MCP-1 levels of COMO patients were significantly higher than those of other groups. We did not find any significant difference in fecal SCFAs among groups (**Supplementary Table 1**).

**Table 1:** Demographic and clinical symptom of IBS-D, depression, COMO patients and HCs.

	HC(a, n=15)	IBS-D(b, n=22)	Depression (c, n=15)	COMO(d, n=13)	*χ* ^2^ or ANOVA		
					*χ* ^2^/*F*	*P* value	Post Hoc (*P* < 0.05)
Sex^*^ (F/M, n)	10/5	10/12	9/6	7/6	1.792	0.617	—
Age (year)	44.8 ± 2.9	40.7 ± 3.1	42.3 ± 3.0	43.1 ± 3.6	0.314	0.815	—
BMI (kg/m^2^)	24.1 ± 0.7	22.9 ± 0.7	21.3 ± 0.7	22.7 ± 1.1	1.682	0.181	—
**IBS-SSS total score**	12.4 ± 7.7	202.7 ± 15.3	144.7 ± 28.7	284.7 ± 21.5	42.978	<0.001	a/b a/c a/d b/c b/d c/d
Abdominal pain	0.0 ± 0.0	25.2 ± 3.8	17.5 ± 9.9	34.7 ± 6.2	8.235	<0.001	a/b a/c a/d c/d
Onset frequency of abdominal pain	0.0 ± 0.0	42.7 ± 7.0	13.3 ± 9.0	50.8 ± 9.0	11.256	<0.001	a/b a/d b/c c/d
Abdominal bloating	0.0 ± 0.0	19.1 ± 4.5	18.8 ± 8.0	49.1 ± 8.0	12.556	<0.001	a/b a/c a/d b/d c/d
Satisfaction with bowel habit	6.1 ± 3.8	64.7 ± 3.4	55.2 ± 8.1	76.4 ± 6.2	39.5	<0.001	a/b a/c a/d c/d
Interference with quality of life	6.3 ± 3.9	50.2 ± 4.8	44.7 ± 10.5	73.8 ± 6.7	20.494	<0.001	a/b a/c a/d b/d c/d
Maximum bowel movements per day	1.7 ± 0.2	2.9 ± 0.3	2.4 ± 0.5	2.8 ± 0.5	2.116	0.108	—
**SDS score**	28.7 ± 1.4	39.5 ± 2.1	58.2 ± 2.3	64.5 ± 3.6	42.526	<0.001	a/b a/c a/d b/c b/d

BMI, Body mass index; IBS-SSS, Irritable bowel syndrome severity scoring system; SDS, Zung self-rating depression scale; —, No comparability; *Comparison of sex ration was performed by χ2 test, others by ANOVA.

### Functional Genomic Analyses of Fecal Microbiota in IBS-D, Depression, COMO Patients, and Healthy Controls

We binned whole-genome sequencing data and conducted taxonomic analysis, aiming to characterize the microbial taxonomic composition in a finer resolution. Non-surprisingly, case-control comparative analysis and biodiversity analysis further proved our previous finding and lead to no novel finding (**Supplementary Table 2**).

To distinguish the microbiota composition functionally, MWAS was performed on the same fecal samples as recorded in our 16S publication (Liu et al., 2016) of each subject (n = 65). A total of 7,816,170 contigs were assembled, with an average length of 2,031 bps (N50), ranging from 2,284 to 26,866 bp per sample. A total of 18.7 million gene sequences were predicted from the contigs, approximately 76.8% of which were identified and assigned to 4,631 functional units (COG) and 26 categories (Altschul et al., 1990; Tanca et al., 2017). Of these genes, an average of 38.4% were related to metabolism, 26.9% to cellular processes and signaling, and 22.8% to information storage and processing (**Supplementary Figure 1**).

**Figure 2:**
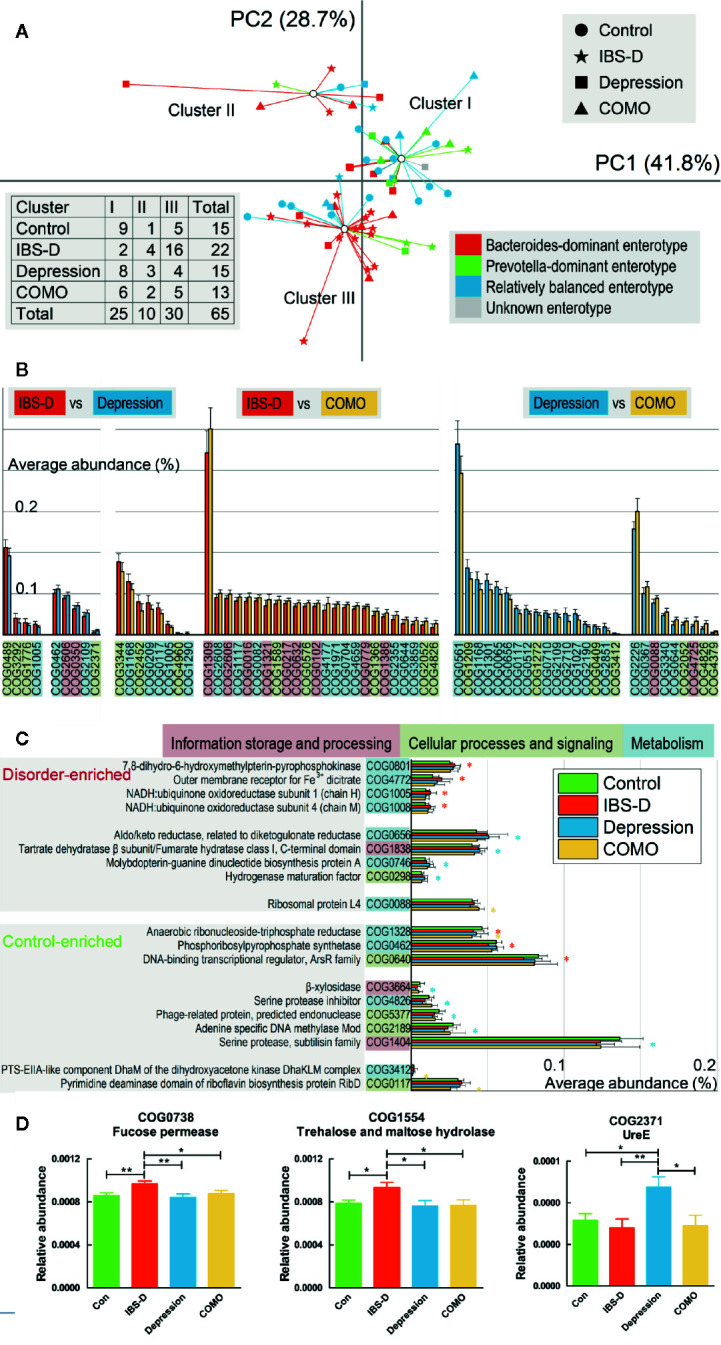
Compositions functional genes and enriched COGs observed in each patient group. **(A)** Scatter plots of 65 baseline individuals by PCA and PAM. The shapes of the dots correspond to different study groups, and the colors correspond to three subtypes identified in our previous study (one individual was not involved in previous study and is denoted as unknown). The first and second principal components accounting for more than 70% of the eigenvalues, and the individuals formed three clusters based on functional compositions. Numbers of patients in each cluster and study group are recorded in the table on the left lower side. **(B)** The abundance of functional genes significantly differed between each of the two disorder groups (p < 0.005). Error bars show standard deviations. Poorly characterized functions are not shown. The background color of each function denotes its class. **(C)** The abundance of functional genes significantly differed between each disorder group and the control group. Error bars show standard deviations. The asterisk (*) indicates a functional gene that was significantly enriched or rare in the corresponding disorder group compared with the control group (p < 0.005). Poorly characterized functions are not shown. The background color of each function denotes its class. For **(B**, **C)**, the background color of each gene denotes its general functional class (a. information storage and processing, b. cellular processes and signaling, c. metabolism, and d. poorly characterized). **(D)** COG0738 and COG1554 were more abundant in IBS-D patients than in all other groups, whereas COG2371 was more abundant in depression patients than in all other groups. In this figure, * represents P value <0.05 and ** represents P value <0.01.

Three functional clusters were identified using PCA and PAM analysis (**Figure 2A**). By contrast to our previous subtypes (Liu et al., 2016) where patients with IBS-D and depression exhibited similar types and the controls exhibited a different type, more than half of the controls (9/15) and patients with depression (8/15) were now in the same cluster (Cluster I), while most IBS-D patients were in another (16/22, Cluster III). These results suggest that individuals with similar microbial enterotype at the genus level may display different functional signatures. For depression and COMO, the proportions of clusters I, II, and III were similar (8/15, 3/15, 4/15 and 6/13, 2/13, 5/13, respectively). Of all functional genes identified, 782 were found to be significantly varied across sample groups (FDR adjusted *P <*0.05; **Supplementary Table 3**). The ones that differed dramatically (W-rank sum test, FDR adjusted *P <*0.005) between each of the two disorder groups or between disorder and control groups are shown in **Figures 2B, C**.

Because the data were not normally distributed, permutation one-way ANOVA was performed (**Supplementary Table 4**) to identify genes significantly varied in different groups. We found that, compared to the other three groups, genes encoding trehalose and maltose hydrolase (COG1554) and fucose permease (COG0738) were significantly enriched in patients with IBS-D. UreE (COG2371) was significantly enriched in patients with depression (**Figure 2D**). To validate our findings, we aligned sequenced genes against the KEGG database to generate an alternative set of annotations. Again, using permutation one-way ANOVA, we identified KEGG-annotated function groups that differed in abundance across study groups (**Supplementary Table 5**). K02429 (MFS transporters, FHS family, L-fucose permease) was significantly different across sample groups (FDR adjusted *P <*0.01), with the highest abundance in IBS-D, consistent to our findings regarding COG0738. Another protein, K03187 (urease accessory protein), also was differed significantly across sample groups (FDR adjusted *P <*0.01), with highest abundance in the depression group, consistent with our findings regarding COG2371.

To identify the microbial species that are potentially responsible for the production of these proteins, we aligned genes annotated with corresponding functions against the RefSeq database of NCBI with PhymmBL (Brady and Salzberg, 2009). We found that the main contributing species for COG0738 and COG1554 included *Escherichia coli*, *Coprococcus catus*, *Bacteroides fragilis* and *Desulfomicrobium baculatum* etc, and for COG2371, included *Escherichia cloacae*, *Coprococcus catus*, *Bacteroides fragilis*, and *Desulfomicrobium baculatum* etc. (**Supplementary Table 6**). Among those species, *E. coli* and *E. cloacae* are two distinct drivers for IBS and depression separately. Literature review of *E. coli* revealed that this species has been frequently reported as associated with IBS (Sobieszczanska et al., 2007; Rajilic-Stojanovic et al., 2011; Bhattarai et al., 2017). Though we could find any research reported that *E. cloacae* linking to depression but *E. cloacae* possesses urease activity (Osaki et al., 2008). Nonetheless, substantially more work is required to determine whether these bacteria could mediate the manipulation of human physiological conditions.

### Genetic Network of Microbial Functions Differ Across Study Groups

**Figure 3:**
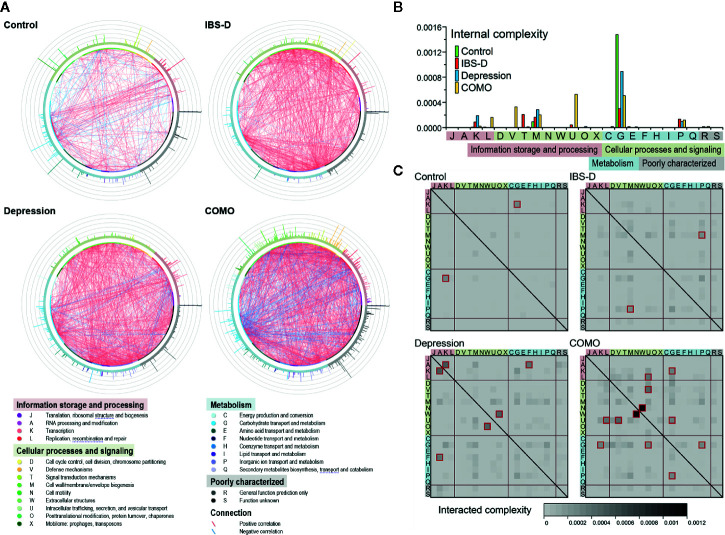
Functional co-occurrence networks and complexity measurement of the functional categories. **(A)** The nodes (bubbles) of the functions are colored according to the functional categories and are in the same order in each group (starting at 3 o’clock, the functions are listed in categories J, A, and K in the counterclockwise direction). The color strip around the nodes corresponds to the four functional classes shown in the legend below. The connections in red or green represent positive (r ≥ 0.8 and q < 0.05) or negative (r ≤ -0.8 and q < 0.05) correlations, respectively. The bars on the periphery show the active indices of the corresponding functions in each network. **(B)** Internal complexity of each category. The color of the columns represents groups as shown in the legend. The background color of each category denotes its class. **(C)** Interaction complexity between each of the two categories. The heat map color shows the interaction complexity between the two corresponding categories. Values higher than 0.0002 are outlined in dark red. The background color of each category denotes its class.

It has been long recognized that genes work collectively to achieve complex functions; therefore, studying gene networks is an essential step in understanding complex biological activities (Thompson et al., 2015). To elucidate the intricate functional associations within microbial community, functional co-occurrence networks were established for each study cohort. In total, 653 functions and 4127 associations (441 in healthy controls, 933 in IBS-D patients, 1,171 in depression patients and 1,582 in COMO patients) were enrolled as nodes and edges in the co-occurrence networks (**Figure 3A**). The healthy controls had the fewest associations among gene microbial functions. More positive than negative correlations were observed between functions in all subjects (372 vs. 69 in the control group, 916 vs. 17 in IBS-D patients, 1,051 vs. 120 in depression patients, 1,272 vs. 310 in COMO patients). Internal and interaction complexities of the functional categories were calculated (**Figures 3B, C**, see detail in *Materials and Methods* section). The category responsible for carbohydrate transport and metabolism (category G), which is essential for a healthy gut microenvironment (Tanca et al., 2017), showed the highest internal complexity in the control group (**Figure 3B**). Because gene modules with more genetic interactions tend to be more active (Zotenko et al., 2008), this observation suggested that category G was a relatively more active category. The disease groups, by contrast, displayed higher association between categories responsible for cell wall/membrane/envelope biogenesis (M) and category of inorganic ion transport and metabolism (P). Moreover, unique associations were observed in some disorder groups. For example, there were associations between functions responsible for signal transduction mechanisms (T) in IBS-D patients, for transcription (K) in depression patients, and for replication, recombination and repair (L), defense mechanisms (V), and intracellular trafficking, secretion, and vesicular transport (U) in COMO patients.

### Non-Targeted Profiling of Host Serum Level Metabolites

**Figure 4:**
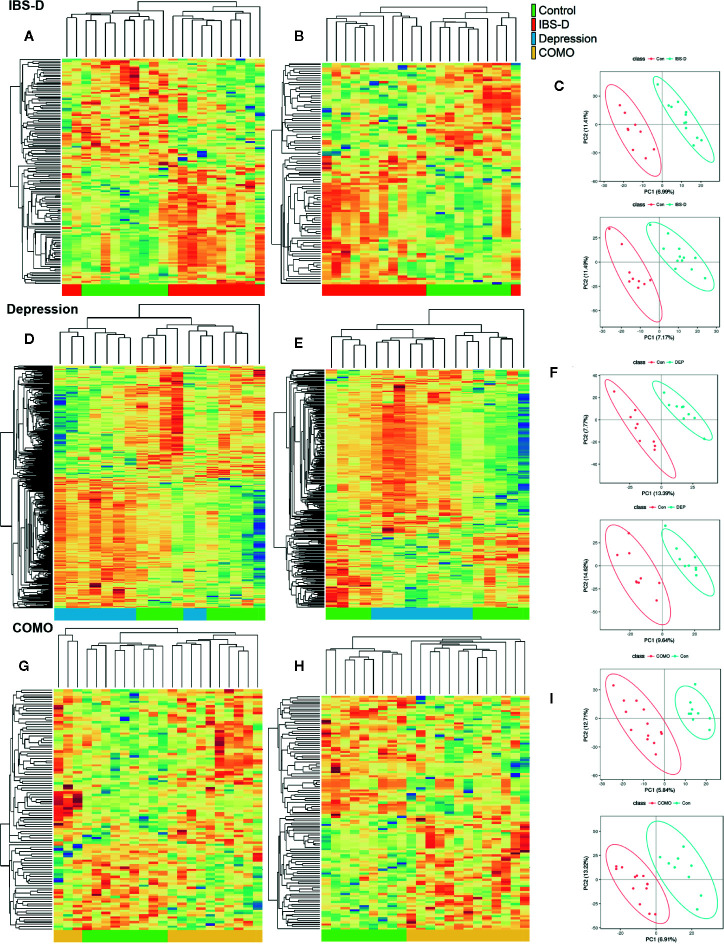
Serum metabolomics by study groups. Cluster analysis of the selected differential ions for the serum metabolome of IBS-D **(A–C)**, depression **(D–F)** and COMO **(G–I)** patients compared with healthy controls. The left columns show negative ions, the middle columns show positive ions and the right columns show PLSDA **(A, B, D, E, G, H)**. Each row in the panel represents a differential ion. Each column represents a sample. Different colors indicate different intensities, and the colors range from green to red, indicating an intensity from low to high. **(C, F, I)** PLS-DA reflects differences between two groups. The abscissa represents the first principal component PC1, and the ordinate represents the second principal component PC2. Each point in the plot represents a sample, and the dispersion of the two colored symbols represents the distribution of the two sets of samples on the PC1 and PC2 axes.

We performed non-targeted metabolomics profiling on serums from patients and controls. IBS-D, depression and COMO patients showed remarkable metabolic alternations compared with healthy controls (**Figure 4**). There were 77 negative and 59 positive compound IDs in IBS-D patients (FDR adjusted *P <*0.05, **Supplementary Tables 7, 8**), 206 negative and 180 positive compound IDs in the depression group (FDR adjusted *P <*0.05, **Supplementary Tables 9, 10**), 54 negative and 71 positive compound IDs in COMO patients (FDR adjusted *P <*0.05, **Supplementary Tables 11, 12**) that were significantly different from healthy controls. Of these, 45 negative and 40 positive compound IDs in IBS-D patients, 105 negative and 123 positive compound IDs in depression, 35 negative and 38 positive compound IDs in COMO patients were upregulated. In the IBS-D group, the changed compounds were mainly associated with carbohydrate metabolic pathways including “fructose and mannose metabolism” and “starch and sucrose metabolism,” amino acid metabolism pathways, including “tyrosine metabolism,” “tryptophan metabolism,” and “arginine and proline metabolism.” Other pathways associated with these metabolites were “arachidonic acid metabolism,” “primary bile acid biosynthesis,” and “citrate cycle (TCA cycle).” In the depression group, the changed metabolites were mainly associated with “arachidonic acid metabolism,” “neuroactive ligand-receptor interaction,” and “Parkinson’s disease.” In addition, the metabolites were also linked to carbohydrate metabolism including “galactose metabolism” and “pentose and glucuronate interconversions.” In the COMO group, the changed metabolites were associated with “vitamin B6 metabolism” and “adrenergic signaling in cardiomyocytes” in addition to the metabolic pathways described above.

### Disease-Enriched COGs and Altered Host Metabolites in IBS-D, Depression, and COMO Patients Correlate With Clinical Indices

Taking from the metabolomics study, COG0738 (fucose permease) and COG1554 (trehalose and maltose hydrolase) were significantly enriched in patients with IBS-D, both of which were functional proteins related to saccharides metabolism. Metabolites related to starch and sucrose metabolism, galactose metabolism, fructose and mannose metabolism pathway were also changed in patients with IBS-D (**Supplementary Tables 7, 8**). As for depression patients, a significant change was observed in metabolites related to the neuroactive ligand-receptor interaction pathway (**Supplementary Tables 9, 10**).

**Figure 5:**
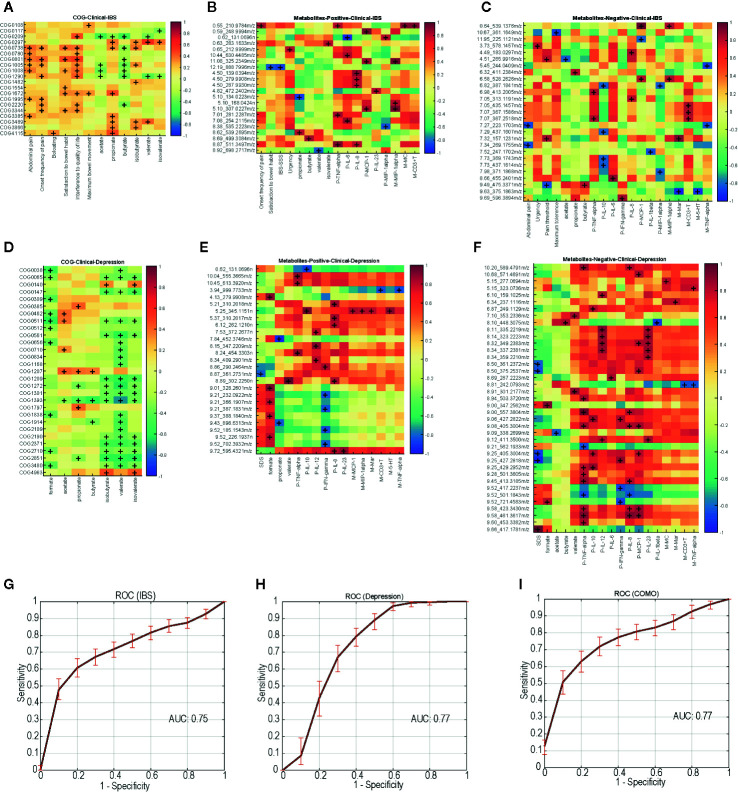
Disease-enriched COGs and altered host metabolites in IBS-D and depression patients associate with clinical indices, ROC curves of the random forest models trained with the profile of functional biomarkers. **(A)** IBS-D enriched COGs associate with clinical indices. IBS-D altered host metabolites included negative ions **(B)** and positive ions **(C)** associate with clinical indices. **(D)** Depression enriched COGs associate with clinical indices. Depression altered host metabolites included negative ions **(E)** and positive ions **(F)** associate with clinical indices. +, p<0.05. **(G–I)** show the performance of discrimination models trained with random forest and the functional biomarkers during five-fold cross validation. The solid lines represent the average ROC curve, and the shadow shows the ROC ranges during 1000 times bootstraps. The average AUC for the model distinguishing IBS from other samples is 0.75, which is 0.77 for depression and 0.77 for COMO model.

To assess a direct correlation between these functional proteins and clinical manifestation, we calculated the covariation between disease-enriched COGs vs. clinical indices, and host metabolites vs. clinical indices. In IBS-D patients, COG0738 (fucose permease) positively correlated with fecal propionate and isobutyrate. COG 1554 positively correlated with onset frequency of pain and bloating (**Figure 5A**). As for host clinical indices, metabolites involved in glycerolipid metabolism and citrate cycle (TCA cycle) pathway positively correlated with onset frequency of pain and urgency. Metabolites in glycerophospholipid metabolism and glycine, serine and threonine metabolism pathway positively correlated with serum TNF-α and IL-6 levels, while metabolites in tyrosine metabolism pathway positively correlated with serum IL-8 levels. Metabolites in galactose metabolism pathway negatively correlated with mucosal MIP-1α and serum MCP-1 levels (**Figures 5B, C**). Similarly, in depression patients, COG2371 (urease accessory protein UreE) negatively correlated with fecal SCFAs levels (**Figure 5D**). Metabolites in neuroactive ligand-receptor interaction pathway negatively correlated with SDS. Metabolites in arachidonic acid metabolism pathway positively correlated with serum IL-12 and IL-23 levels (**Figures 5E, F**). The correlations of COMO patients are shown in **Supplementary Figure 2**.

### Biomarkers

To test whether the microbial functions can serve as prediagnostic biomarkers, we trained random forest models using the profile of those specific functions (biomarkers) with significantly different abundance in different groups (permutational one-way ANOVA *P <*0.01 with 1000 randomizations) and also correlated with clinical indices (Spearman correlation with *P <*0.05, Bonferroni-corrected). For IBS-D, depression and COMO, we separately trained the model using biomarkers with highest abundance in the corresponding case group (**Supplementary Table 13**). The models were trained to distinguish one case from the others using five-fold cross validation. The results returned 13, 8, and 34 COGs as biomarkers for IBS-D, depression and COMO, respectively. Biomarkers for IBS-D and depression also included COG0738, 1554 and 2371. The model performance was evaluated using average area under ROC curves (AUC) using 100 times of permutations of five-fold cross validation (**Figures 5G–I**). The average AUC of the model distinguishing IBS-D from others was 0.75, the average AUC of the depression discriminative model was 0.77, and that of the COMO discriminative model was 0.77. This result suggests the discriminatory power of these functional biomarkers.

## Discussion

Several evidences suggest that the gut microbiota is important for the pathogenicity of diseases (Dethlefsen et al., 2007). IBS-D and depression have similar structures in the gut microbiota, and the high comorbidity of depression and IBS-D provides further evidence for the brain–gut axis (Cryan and Dinan, 2012). These findings suggest that characterizing gut microbiota related to the pathogenesis of IBS-D, depression and COMO, and identifying corresponding therapeutic targets are pivotal next steps.

We performed metagenome sequencing and characterized all fecal microbiota genes into three clusters among IBS-D, depression and COMO patients. The functional genes for carbohydrate transport and metabolism were essential in the healthy network, whereas those for cell wall/membrane/envelope biogenesis, inorganic ion transport and metabolism could be involved in disordered networks when bacteria invade host cells. Of these, COG0738 (fucose permease) and COG1554 (trehalose and maltose hydrolase) were significantly enriched in IBS-D patients and COG2371 (UreE) in depression patients. The findings of these functional genes were again confirmed when aligning sequences against the KEGG database.

COG0738 encodes fucose permease, which was reported to be associated with bacterial conglutination and invasion against host cells (Stahl et al., 2011). Fucose is a component of mucin in the gut mucosal barrier (Tailford et al., 2015). Fucose permease is positively related to the consumption of host mucin from gut mucus. Mucin glycan and its catabolism participate in bacterial community and colonization. Unexpected alterations of mucin-degradation interrupt gut homeostasis (Park et al., 2018). The enrichment of fucose permease may lead to mucin degradation in the gut mucosa and destroy gut barrier function, characteristic of pathological alteration in IBS-D. Trehalose and maltose hydrolase encoded by COG1554 are involved in the glycolysis pathway for trehalose and maltose that are not absorbed in the small intestine; their products, glucose and gas, can increase gut osmotic pressure (Cardoso et al., 2007), resulting in diarrhea and bloating, both of which are typical symptoms of IBS-D. We observed that COG 1554 positively correlated with onset frequency of abdominal pain and bloating in IBS-D patients. Interestingly, the abundance of COG1554 was significantly lower after treatment with Bifico (Bifico^®^ [SINE, Shanghai, CHN]) in the COMO (IBS-D and depression comorbidity) patients (data not shown), whose abdominal symptoms were also significantly improved (Zhang et al., 2019).

On the other hand, the genes encoding urease accessory proteins, UreE (COG2371), was significantly enriched in patients with depression relative to control and IBS-D groups; the protein acts as a metallochaperone, delivering Ni ions and activating the urease (Mulrooney et al., 2005), allowing bacteria to utilize urea for ammonia production. Ammonia plays an important physiological role because it provides usable forms of nitrogen required for the synthesis of DNA, RNA, and proteins (Liu et al., 2017). Fat-soluble ammonia can enter the brain through the blood-brain barrier and can be used with glutamic acid to synthesize glutamine, leading to a decrease in the excitatory neurotransmitter. This pathway is thought to be a potential mediator for psychiatric disorders such as depression (Duan et al., 2015).

We then searched for the bacterial species responsible for these COGs. *E. coli* was determined to mainly contribute to COG0738 and COG1554, while *E. cloacae* was determined to contribute to COG2371. *E. coli* is a conditional pathogen that is commonly related with diarrhea (Roubos-van et al., 2017; Alizade et al., 2019) and is involved in the pathogenesis of IBD (O’Brien et al., 2017; Palmela et al., 2018), especially the adherent-invasive *E coli* (AIEC) pathotype. The uptake of L-fucose (Dang et al., 2010) is one of the carbon sources for *E. coli*, as is maltose (Baev et al., 2006). *E. cloacae* possesses urease activity (Osaki et al., 2008), which can hydrolyze urea into ammonia.

We found out COG0738 and COG1554 was enriched in patients with IBS-D and sucrose and fructose metabolism pathway of host metabolism also changed in the same subjects. Similar in patients with depression, COG2371 was enriched and neuron related host metabolism also altered. We supposed that different functional genes of gut microbiota can affect host clinical manifestations through the alteration of host metabolites, we tried to search their potential relationship by multiple correlative analyses in order to find out the relationships between COGs and diseases.

**Figure 6:**
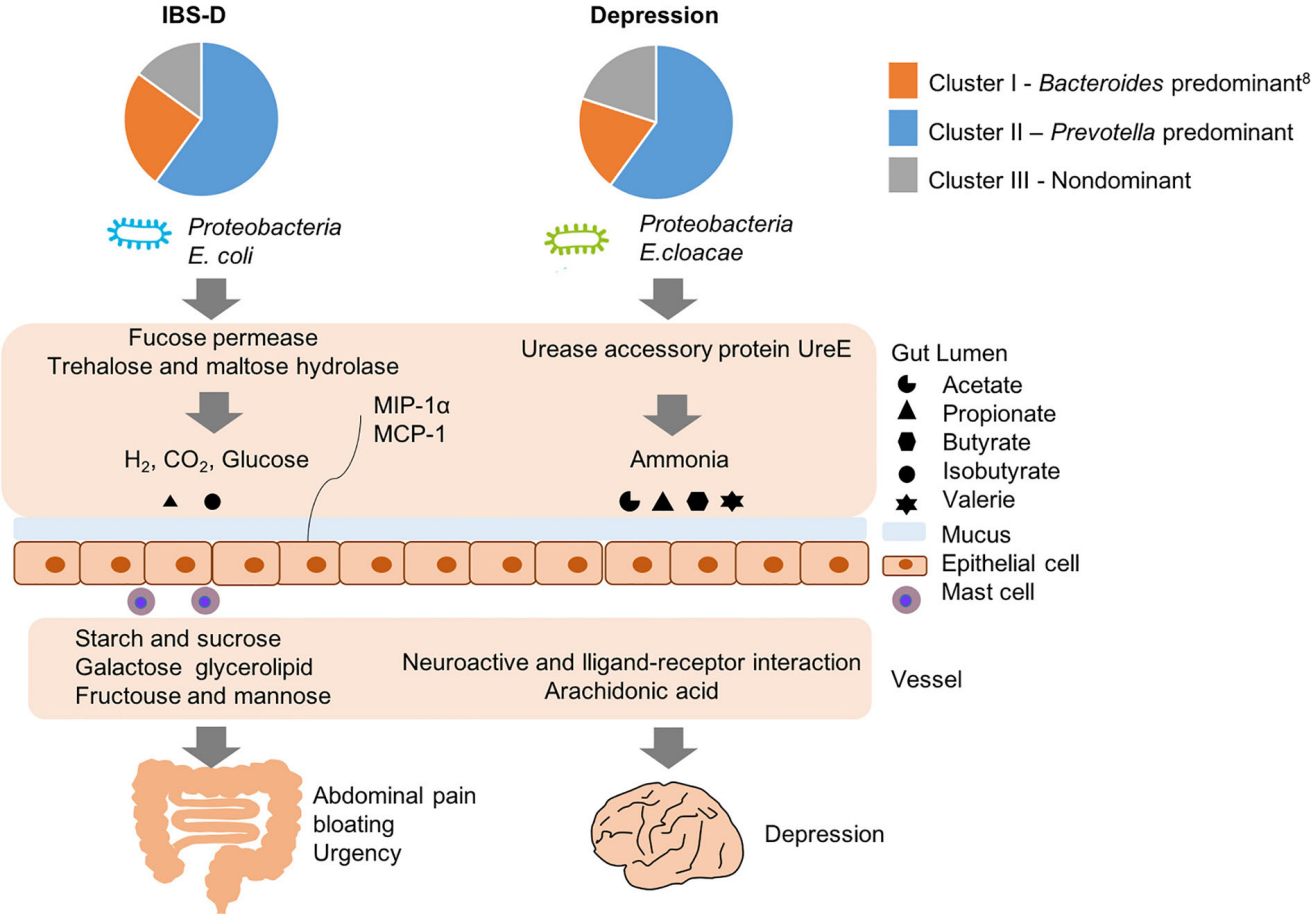
Potential pathogenesis of IBS-D and depression with the similar structure of gut microbiome but different functions.

We attempted to identify an inner correlation to explain why similar fecal structures of IBS-D and depression manifest different pathologies, and here we propose several potential pathogenic pathways (**Figure 6**): for IBS-D, *E. coli* hydrolyzed trehalose and maltose *via* trehalose and maltose hydrolase into glucose and gas such as hydrogen and carbon dioxide, resulting in abdominal pain, bloating and diarrhea. *E. coli* could also consume mucin from gut mucus *via* fucose permease, leading to damage the function of gut barrier (Chang et al., 2004; Tenaillon et al., 2010). By contrast, when *E. coli* are grown anaerobically, they perform mix-acid fermentation, producing acetate, ethanol, lactate, formate and succinate as major products (Li et al., 2017). Enrichment of fucose permease and trehalose and maltose hydrolase might cause clinical manifestation of IBS-D through formate, acetate and mucosal inflammation. Likewise, for depression, *E. cloacae* possesses urease activity that hydrolyzes urea into ammonia; ammonia is toxic for the human central nervous system and can cause psychological disturbances and behavioral disorders such as depression. On the other hand, we found *E. cloacae* also correlated with alterations in host metabolism of neuroactive ligand-receptor interaction and arachidonic acid metabolism. Neuroactive ligand-receptor might include dopamine receptor, γ-aminobutyric acid (GABA) receptor and N-methyl-D-aspartate receptor, eventually contributing to the onset of depression. Furthermore, higher serum arachidonic acid/eicosapentaenoic acid ratios are associated with greater likelihood of depressive symptoms in some Japanese subjects (Shibata et al., 2018) and the metabolite in arachidonic acid metabolism is higher in depression patients than healthy controls.

Our research has some limitations. First, the correlation of products of functional genes and the host metabolites requires further research. Second, the question as to whether COMO derives from metabolism or correlation analysis is not resolved by simple superposition of IBS-D on depression; this requires further study. Third, the mechanism of pathogenic for *E. coli* and *E. cloacae* in IBS-D and depression requires further verification. Despite these limitations, the study as a whole suggests that the disease-related functional transformation of human gut microbiome is significant and meaningful.

## Conclusions

IBS-D and depression share similar fecal microbiome signatures but show diverse clinical manifestations, likely due to differences in microbial functions. *E. coli* may contribute to IBS-D through fucose permease and trehalose and maltose hydrolase. *E. cloacae* produces ammonia through UreE, resulting in symptoms of depression. We captured those functions with significantly different abundance and also identified potential prediagnostic biomarkers. Our findings provide insights into the involvement of gut microbiota in diseases related to brain–gut disorder and provide novel targets for treatment of IBS-D and depression.

## Data Availability Statement

The original contributions presented in the study are publicly available. This data can be found here: https://www.ebi.ac.uk/ena/browser/view/PRJEB40628 PRJEB40628.

## Ethics Statement

The studies involving human participants were reviewed and approved by The Ethics Committee of Peking University Health Science Center (No. 2013-112). The patients/participants provided their written informed consent to participate in this study.

## Author Contributions

Conceiving and design: LD. Supervising of study and critical revision of manuscript: LD and HZ. Subject recruitment and performance of clinical procedure: LZ, YL, JJZ, XiangW, KW. Bioinformatics analysis: CX, ZW, XiaoW, ML. Performance of metabolomics profiling and data analysis: QJ, SZ, JDZ, QS. Drafting of manuscript: QJ, CX, LZ, ZW. Corresponding authors: LD and HZ. All authors contributed to the article and approved the submitted version.

## Funding

The studies were supported by “National Twelfth Five-Year Plan for Science and Technology of China (2012BAI06B02)” and “National Natural Science Foundation of China (81670491).”

## Conflict of Interest

The authors declare that the research was conducted in the absence of any commercial or financial relationships that could be construed as a potential conflict of interest.

## Supplementary Material

Supplementary Figure 1 Global overview of the MWGS data. **(A)** Ratio of assembled reads from each group. **(B)** Distribution of functional categories among 65 individuals. Each row denotes a functional category listed on the left and described on the right, each column represents an individual below corresponding to the group on top. The heat map color shows the abundance

Supplementary Figure 2 COMO enriched COGs and altered host metabolites associate with clinical indices. **(A)** COMO enriched COGs associate with clinical indices. COMO altered host serum metabolites included negative ions **(B)** and positive ions **(C)** associate with clinical indices. +: *P*<0.05
