# Supplementary material for: Multiomics Study of Gut Bacteria and Host Metabolism in Irritable Bowel Syndrome and Depression Patients
Source: Front Cell Infect Microbiol. 2020 Oct 29;10:580980. doi: 10.3389/fcimb.2020.580980 (PMC7658686; doi:10.3389/fcimb.2020.580980)
Supplement: Supplementary file 2 [file DataSheet_2.docx]

**Fecal samples collection**

Fecal samples were collected before colonoscopy. Fecal samples were collected by patients at home using a special fecal collection packages (including special paper, stick, a collection tube and icebags). Fecal sample was sealed with a plastic bag, put in a transport bag with ice bag and sent to laboratory within 2 hours. All the fecal samples were stored at -80 ℃ refrigerator until use. **Immunohistochemistry**

Immunohistochemistry staining was performed using the ZSGB-BIO ALK system (ZK-9600, Origene & ZSGB-BIO) on formalin-fixed paraffin-embedded biopsies with primary antibodies against MCP-1 (ab9669, 1:400; Abcam), MIP-1α (sc-33203, 1:400; Santa), TNF-α (ab49579, 1:200; Abcam), 5-HT (ab16007, 1:1000; Abcam), Mast cell (ab2378, 1:400; Abcam), Macrophage (ab49394, 1:80; Abcam), or CD3^+^ T cell (ab699, 1:20; Abcam). As a secondary antibody and for visualization, a peroxidase/3,3-diaminobenzidine (DAB^+^) was used according to manufacturer's protocol (ZSGB-BIO ALK Detection System Peroxidase DAB^+^ Rabbit/Mouse; PV-6000-D, Origene & ZSGB-BIO). MCP-1, MIP-1α, TNF-α, and 5-HT expression levels were evaluated based on staining intensity (SI) and the percentage of positive cells (PP) and scored using the immunoreactive score (IRS): IRS = SI × PP. SI was scored as follows: 0, negative; 1, weak; 2, moderate; and 3, strong. PP was scored as follows: 0, 0-1%; 1, 2-10%; 2, 11-50%; 3, 51-80%; and 4, >80% positive cells. Scores from 0 to 12 points were obtained. Positive cell numbers of mast cells, macrophages and CD3^+^ T cells were quantified as the mean of positive cell number of three random high-power filed (×400) per sample.

**Detection of systematic inflammation using ELISA**

All samples and standards were assayed in duplicates. To start with the measurement, each well of the 96-well plate was prewet with 200 μl assay buffer, then covered with a foil plate sealer and incubated 10 min at room temperature on a shaker. A volume of 25 μl of standard, wash buffer (served as the blank), or sample and 25 μl microparticles was added to each well and incubated at 4 °C overnight. The liquid in each well was removed, and wells were washed with 200 μl wash buffer twice. After wash buffer was removed thoroughly, 25 μl biotinylated antibody was added to each well and incubated at room temperature for 2 hours. The liquid in wells was removed and wells were washed with 200 μl wash buffer twice more. Then, 25 μl streptavidin-phycoerythrin was added to each well and incubated at room temperature for 30 min followed by two washes with wash buffer. A volume of 150 μl wash buffer was added to each well to resuspend to microparticles and incubated for 5 minutes on the shaker. The plates were placed into a Luminex 200 to measure median fluorescence intensity of standards and samples.

### Quantification of fecal SCFAs

100 mg Fecal samples were prepared with 1 ml 50% aqueous acetonitrile, then 40 μl supernatants were collected after centrifugation at 14,000 *g* for 10 min and mixed with 20 μl of 200 mM 3-nitrophehylhydrazine (3NPH) (Sigma-Aldrich) in 50% aqueous acetonitrile and 20 μl of 120 mM N-(3-dimethylaminopropyl)-N´-ethylcarbodiimide (EDC)-6% pyridine (Sigma-Aldrich) solution in the same solvent for derivatization at 40 °C for 30 min. To obtain isotope-labeled internal standards (IS), 50 μl of a mixed standard solution containing 4 mM of acetate, 2 mM of propionate and 1 mM of each of the other five SCFAs (Sigma-Aldrich) were added to a mixture containing 1 mg ^13^C6-3NPH•HCl (QUALITY CONTROL CHEMICALS INC.), 25 μl of 120 mM EDC in 50% aqueous acetonitrile and 25 μl 6% pyridine solution in the same solvent and derivatized under the same condition mentioned above. A mixed standard solution containing 1 mM of each of the seven SCFAs was diluted to have concentrations of 0.2-500 μM and the resulting solutions were used as working standard solutions, and were further derivatized at 40 °C for 30 min.

The Dionex Ultimate 3000 UPLC system was coupled to a TSQ Quantiva Ultra triple-quadrupole mass spectrometer (Thermo Fisher), equipped with a heated electrospray ionization (HESI) probe in negative ion mode. Extracts were separated by a BEH C18 column (2.1×100 mm, 1.7 μm, Waters). A binary solvent system was used, in which mobile phase A consisted of 0.01% formic acid and 100% H_2_O, and mobile phase B of 0.01% formic acid and 100% acetonitrile. An 18-minute gradient with flow rate of 350 μL/min was used as follows: 0–1.5 min at 5% B; 1.5–4 min, 5–15% B; 4–12 min, 15-55% B; 12–13 min, 55–98% B; 13–15 min, 98% B and 15.1–18 min, 5% B. Column chamber and sample tray were held at 40 °C and 10 °C, respectively. Data acquired in selected reaction monitoring (SRM) for each fatty acid. Both of precursor and fragment ion were collected with resolution of 0.7 FWHM, respectively. The source parameters are as follows: spray voltage: 1000 V; ion transfer tube temperature: 350 °C; vaporizer temperature: 450 °C; sheath gas flow rate: 40 Arb; auxiliary gas flow rate: 20 Arb. CID gas: 2.0 mTorr.

### DNA extraction, library construction and sequencing

The microbial community DNA was extracted using MagPure Stool DNA KF kit B(Magen，China) following the manufacturer's instructions. DNA was quantified with a Qubit Fluorometer by using Qubit dsDNA BR Assay kit (Invitrogen, USA) and the quality was checked by running aliquot on

1% agarose gel.

After DNA extraction, 1 µg genomic DNA was randomly fragmented by Covaris, followed by purification by AxyPrep Mag PCR clean up k it. The fragmented DNA was selected by Agencourt AMPure XP Medium kit to an average size of 200 400bp. The fragments were end repaired by End Repair Mix and purified afterwards. The repaired DNAs were combined with A Tailing Mix, then the Illumina adaptors were ligated to the Adenylate 3’Ends DNA and followed by purification. The products were selected bas ed on the insert size. Several rounds of PCR amplification with PCR Primer Cocktail and PCR Master Mix were performed to enrich the Adapter ligated DNA fragments. After purification, the library was qualified by the Agilent 2100 bioanalyzer (Agilent, USA) and ABI StepOnePlus Realtime PCR System. Finally, the qualified libraries were sequenced on Illumina Hiseq platform (BGI Shenzhen, china).

### Non-target metabolomics

Firstly, all chromatographic separations were performed using an ultra-performance liquid chromatography (UPLC) system (Waters). An ACQUITY UPLC BEH C18 column (100 mm x 2.1 mm, 1.7 μm, Waters) was used for the reversed phase separation. The column oven was maintained at 50 °C. The flow rate was 0.4 ml/min and the mobile phase consisted of solvent A (water + 0.1% formic acid) and solvent B (acetonitrile + 0.1% formic acid). Gradient elution conditions were set as follows: 0–2 min, 100% phase A; 2–11 min, 0% to 100% B; 11–13 min, 100% B; 13–15 min, 0% to 100% A. The injection volume for each sample was 10 μl.

A high-resolution tandem mass spectrometer Xevo G2 XS QTOF (Waters) was used to detect metabolites eluted form the column. The Q-TOF was operated in both positive and negative ion modes. For positive ion mode, the capillary and sampling cone voltages were set at 3 kV and 40 V, respectively. For negative ion mode, the capillary and sampling cone voltages were set at 1 kV and 40 V, respectively. The mass spectrometry data were acquired in Centroid MSE mode. The TOF mass range was from 50 to 1200 Da and the scan time was 0.2 s. For the MS/MS detection, all precursors were fragmented using 20–40 eV, and the scan time was 0.2 s. During the acquisition, the LE signal was acquired every 3 s to calibrate the mass accuracy. To evaluate the stability of the LC-MS during the whole acquisition, a quality control sample (pool of all samples) was acquired after every 10 samples.

### Synthesis of functional co-occurrence networks

The network was evaluated by several indices to investigate the different functional abnormity of the disorder groups. Suppose that a network is supported by 26 COG categories namely$\left\{X_{m}\right\}_{m=1}^{26}=\left\{A,B,\cdots,Z\right\}$, and $G(X_{m})=\left\{x_{mi}\right\}_{i=1}^{g(X)}$ is a set of functions involved in *X_m_*, where *g*(*X_m_*)=|*G*(*X_m_*)| is the cardinality of *G*(*X_m_*). For any protein *x_mi_*∈*G*(*X_m_*) corresponding to a node of the network, the existence of association between *x_mi_* and another protein *x_nj_*∈*G*(*X_n_*) is given by

$$\delta(x_{mi},x_{nj})=\left\{\begin{matrix} 1, & \mathrm{if}\text{ }\left|r(x_{mi},x_{nj})\right|\geq0.8\text{ }\mathrm{and}\text{ }q(x_{mi},x_{nj})<0.05; \\ 0, & \mathrm{otherwise}, \end{matrix}\right.$$

where *r* and *q* show the correlation coefficient and corresponding *q*-value between the two proteins *x_mi_* and *x_nj_*. The associated protein set of *x_mi_* is defined as the neighbor node set in:

$A(x_{mi})=\left\{\left.x_{nj}\right|x_{nj}\neq x_{mi};\text{ }\delta(x_{nj},x_{mi})=1;\text{ }x_{nj}\in G(X_{n}),\text{ }n=1,2,\cdots,26\right\}$.

Let *a*(*x_mi_*)=|*A*(*x_mi_*)| to be the count of associated proteins of *x_mi_* and$b(x_{mi})=\left|\left\{\left.(x_{n_{1}j_{1}},x_{n_{2}j_{2}})\right|x_{n_{1}j_{1}},x_{n_{2}j_{2}}\in A(x_{mi});\text{ }\delta(x_{n_{1}j_{1}},x_{n_{2}j_{2}})=1;\text{ }j_{1}\neq j_{2}\right\}\right|$

to be the count of associations among proteins in $A(x_{mi})$. The active index of protein *x_mi_* is thus evaluated by a modified definition free from singular point of cluster coefficient^1^:

$$c(x_{mi})=\frac{a(x_{mi})(a(x_{mi})-1)+1}{2b(x_{mi})+1}-1$$

Another two indices for evaluation of functional categories were also worth attention. To measure the internal interaction of category *X_m_*, let

$$v(x_{mi}|X_{m})=\sum_{\begin{matrix} k=1 \\ k\neq i \end{matrix}}^{g(X_{m})} \delta(x_{mi},x_{mk}),\text{ }x_{mi}\in X_{m}$$

denote the vertex degree of protein in *X_m_*, then the internal complexity of association among functional proteins in *X_m_* is measure by $\sum_{i=1}^{g(X_{m})} v(x_{mi}|X_{m})\ln v(x_{mi}|X_{m})$, as a local definition of network complexity. For comparison of different categories, the index gets normalized by its maximum value as

$$t(X_{m})=\frac{\sum_{i=1}^{g(X_{m})} v(x_{mi}|X_{m})\ln v(x_{mi}|X_{m})}{g(X_{m})\left(g(X_{m})-1\right)\ln\left(g(X_{m})-1\right)}$$

Similarly consider the interaction between two functional categories *X_m_* and *X_n_*, and let

$v(x_{mi}|X_{n})=\sum_{j=1}^{g(X_{n})} \delta(x_{mi},x_{nj}),\text{ }x_{mi}\in X_{m}$; $v(x_{nj}|X_{m})=\sum_{i=1}^{g(X_{m})} \delta(x_{nj},x_{mi}),\text{ }x_{nj}\in X_{n}$

The interacted complexity is thus defined as

$$r(X_{m},X_{n})=\frac{\sum_{i=1}^{g(X_{m})} v(x_{mi}|X_{n})\ln v(x_{mi}|X_{n})+\sum_{j=1}^{g(X_{n})} v(x_{nj}|X_{m})\ln v(x_{nj}|X_{m})}{g(X_{m})g(X_{n})\ln\left(g(X_{m})g(X_{n})\right)}$$

**References:**

^1^ Chun, H. H., Spiegel, E. T. & Solomon, I. C., Burst-to-Burst variability in respiratory timing, inspiratory-phase spectral activity, and inspiratory neural network complexity in urethane-anesthetized C57BL/6 mice in vivo. *ADV EXP MED BIOL* **605** 407 (2008).
