# Supplementary figures and images for: Multiomics Study of Gut Bacteria and Host Metabolism in Irritable Bowel Syndrome and Depression Patients
Source: Front Cell Infect Microbiol. 2020 Oct 29;10:580980. doi: 10.3389/fcimb.2020.580980 (PMC7658686; doi:10.3389/fcimb.2020.580980)

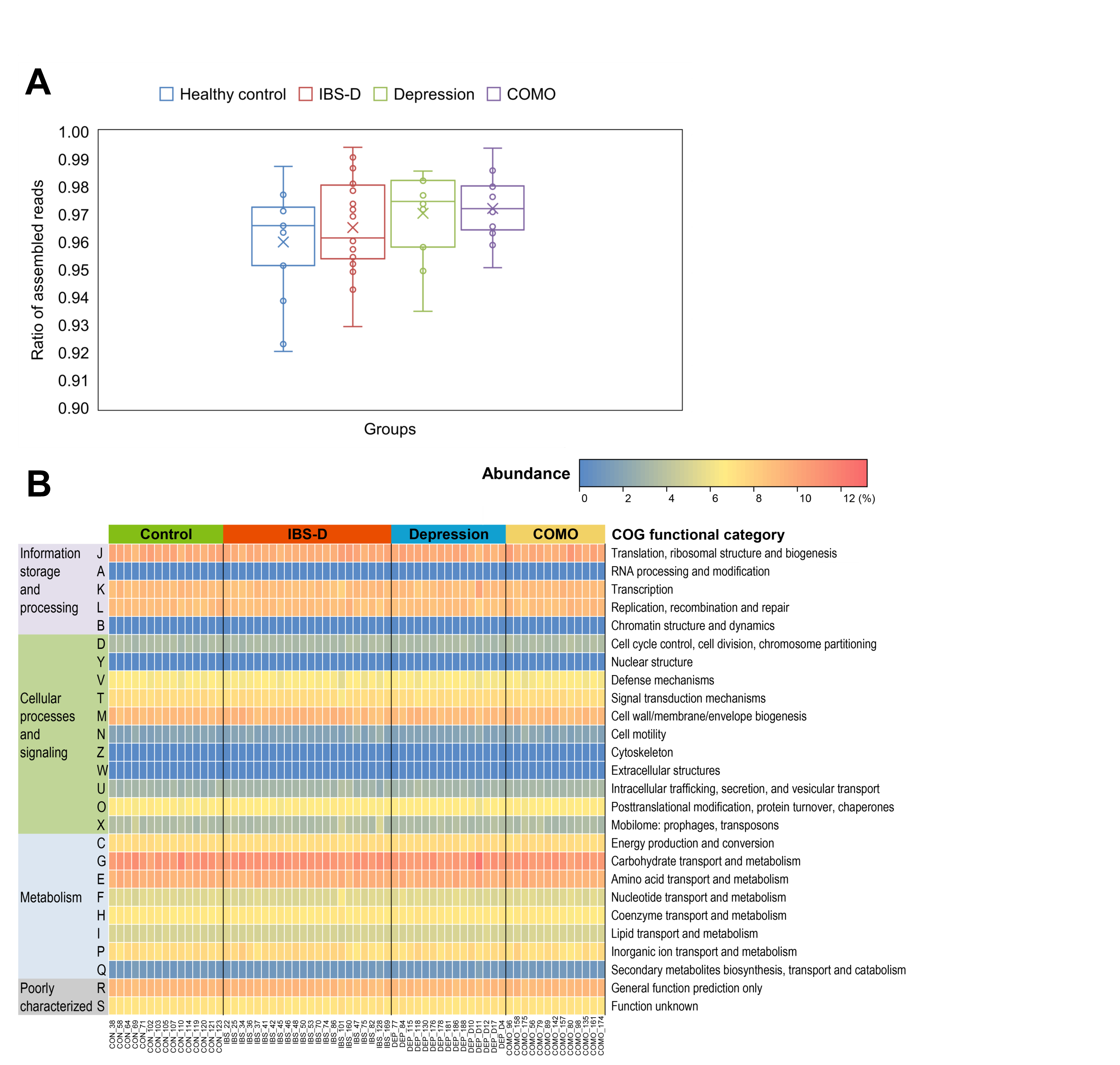

Supplement: Supplementary Figure 1 — Global overview of the MWGS data. (A) Ratio of assembled reads from each group. (B) Distribution of functional categories among 65 individuals. Each row denotes a functional category listed on the left and described on the right, each column represents an individual below corresponding to the group on top. The heat map color shows the abundance [file Image_1.tif]

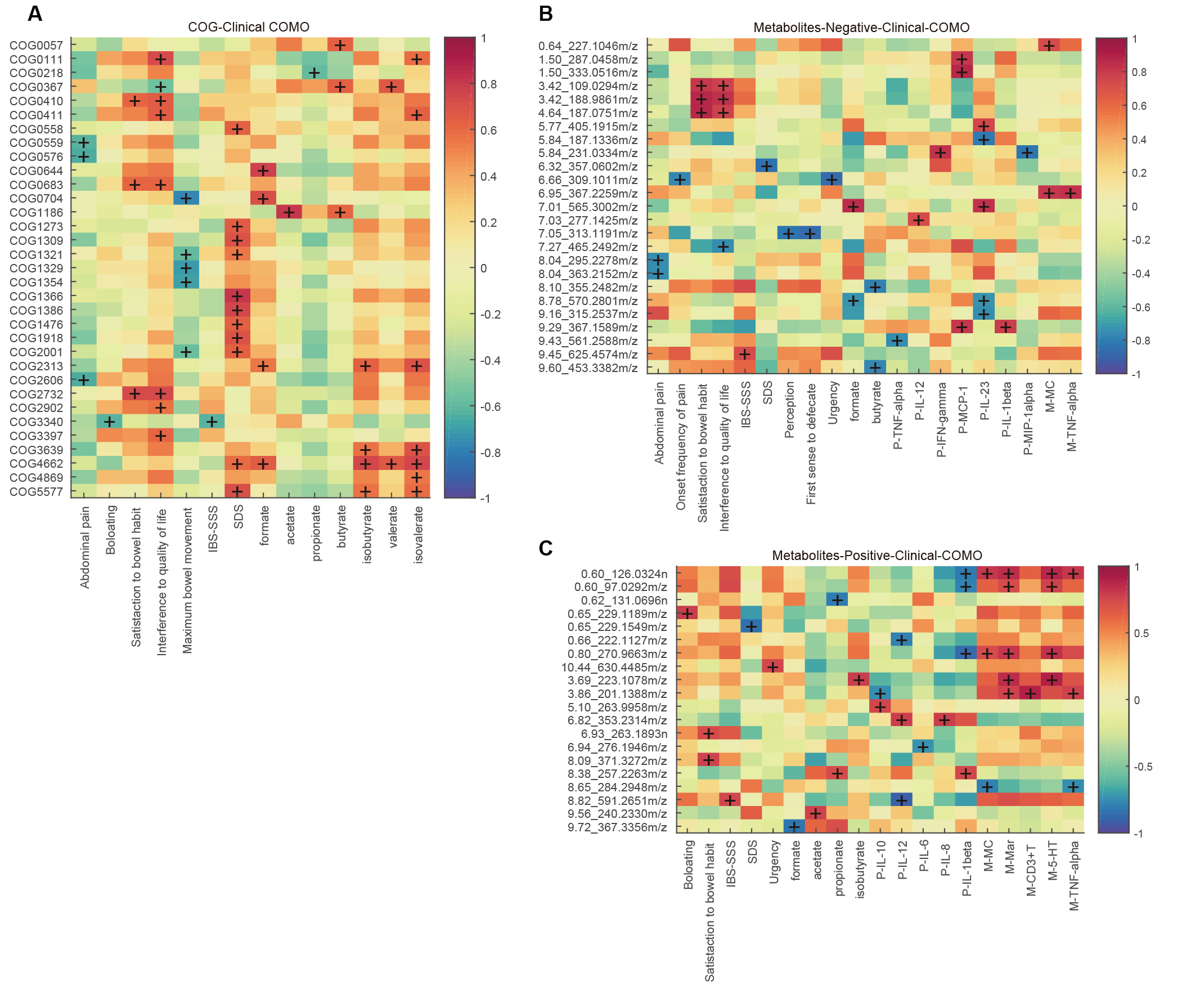

Supplement: Supplementary Figure 2 — COMO enriched COGs and altered host metabolites associate with clinical indices. (A) COMO enriched COGs associate with clinical indices. COMO altered host serum metabolites included negative ions (B) and positive ions (C) associate with clinical indices. +: P<0.05 [file Image_2.tif]
